# Engineering a thalamo-cortico-thalamic circuit on SpiNNaker: a preliminary study toward modeling sleep and wakefulness

**DOI:** 10.3389/fncir.2014.00046

**Published:** 2014-05-20

**Authors:** Basabdatta S. Bhattacharya, Cameron Patterson, Francesco Galluppi, Simon J. Durrant, Steve Furber

**Affiliations:** ^1^School of Engineering, Engineering Hub, University of LincolnLincoln, Lincolnshire, UK; ^2^School of Computer Science, APT Group, University of ManchesterManchester, Lancashire, UK; ^3^School of Psychology, Lincoln Sleep and Cognition Laboratory, University of LincolnLincoln, Lincolnshire, UK

**Keywords:** SpiNNaker, thalamo-cortico-thalamic circuit, computational model, sleep, Izhikevich model, synaptic connectivity, PyNN

## Abstract

We present a preliminary study of a thalamo-cortico-thalamic (TCT) implementation on SpiNNaker (Spiking Neural Network architecture), a brain inspired hardware platform designed to incorporate the inherent biological properties of parallelism, fault tolerance and energy efficiency. These attributes make SpiNNaker an ideal platform for simulating biologically plausible computational models. Our focus in this work is to design a TCT framework that can be simulated on SpiNNaker to mimic dynamical behavior similar to Electroencephalogram (EEG) time and power-spectra signatures in sleep-wake transition. The scale of the model is minimized for simplicity in this proof-of-concept study; thus the total number of spiking neurons is ≈1000 and represents a “mini-column” of the thalamocortical tissue. All data on model structure, synaptic layout and parameters is inspired from previous studies and abstracted at a level that is appropriate to the aims of the current study as well as computationally suitable for model simulation on a small 4-chip SpiNNaker system. The initial results from selective deletion of synaptic connectivity parameters in the model show similarity with EEG power spectra characteristics of sleep and wakefulness. These observations provide a positive perspective and a basis for future implementation of a very large scale biologically plausible model of thalamo-cortico-thalamic interactivity—the essential brain circuit that regulates the biological sleep-wake cycle and associated EEG rhythms.

## 1. Introduction

Computational models are being adopted at an increasing rate as a tool to investigate the cellular mechanisms of brain rhythms in both normal and pathological conditions (Aradi and Érdi, 2006; Breakspear et al., 2010; Terry et al., 2011). While computational resource is an obvious constraint in such endeavors, two further significant obstacles in mimicking the biology are parallelizing neuronal activity, and “de-syncing” the population activity from the master-clock of the computer. Our longer-term interest is in mimicking electroencephalogram (EEG) signatures of the sleep-wake cycle, by simulating biologically plausible computational models using biologically plausible computational techniques. In recent years the University of Manchester has been developing SpiNNaker (Spiking Neural Network architecture), a bespoke massively parallel machine to mimic the inherent parallelism of neuronal activity in real time (Furber et al., 2013). The brain-inspired parallel and asynchronous architecture of SpiNNaker permits biologically plausible computation of brain models—a feature that would otherwise rely on heavyweight software and its compilation on conventional Von-Neumann architectures, and yet achieve minimal parallelism. The study presented here is an initial attempt to design and implement a thalamo-cortico-thalamic (TCT) circuitry on the intrinsically parallel SpiNNaker, which can then be scaled up to mimic biologically plausible EEG signatures of the sleep-wake cycle. The purpose of this work is to demonstrate, as a proof of concept, that such a model can be implemented on SpiNNaker, and to investigate the benefits and drawbacks of this approach. It is not our intention here to produce a model which fully and correctly replicates all brain rhythms measured by EEG in regard to the TCT circuitry; capturing the complex dynamics involved in that system is beyond the scope of the current work.

Neuronal dynamics recorded in EEG, often termed brain rhythms (Buzsáki, 2006), are an inexpensive and popular means of correlating brain activity with its various functional states (Wright and Liley, 1996; Nunez, 2000). The feed-forward and feed-back circuitry between the thalamus and the cortex has long since been known to play a key role in modulating brain rhythms associated with the various sleep stages as well as the sleep-wake transition (Steriade et al., 1993; Steriade, 2003, 2005; Crunelli et al., 2011). Computational models of the TCT brain circuit have therefore been the basis for studying neuronal mechanisms in sleep (Lumer et al., 1997a; Hill and Tononi, 2005; Traub et al., 2005; Bojak et al., 2011; Olbrich et al., 2011; Robinson et al., 2011) as well as in conditions where the EEG is qualitatively similar to certain sleep stages such as epilepsy (Breakspear et al., 2006) and under anaesthesia (Hutt and Longtin, 2010). While all such models refer to a similar holistic structure of the thalamocortical circuit, the models' internal structure, simulation platforms and parameterizations are significantly diverse. Thus, a fundamental aspect in computational modeling of the brain is the level of abstraction; the level of biological detail incorporated in a model needs to be appropriate to the problem at hand. For example, Olbrich et al. (2011) has attempted a multi-scale (time) model architecture in sleep, while (Bojak et al., 2011) has stressed on multi-modal models. On the other hand, (Hill and Tononi, 2005) have based their model on that of Lumer et al. (1997a,b) and have looked into a multi-columnar model of the thalamocortical circuit to mimic brain rhythms of sleep and wakefulness as well as to understand memory consolidation during sleep (Nere et al., 2013).

Another key aspect is the source of experimental data for both model structure and parameterizations. Comprehensive data on synaptic connectivity in the mammalian visual cortex is available in the works of Binzegger et al. (2004); Douglas and Martin (2004) and Neymotin et al. (2011) with some estimation for parameters which were not available from physiological studies. Further, extensive physiological data on rodent and other mammalian lateral geniculate nucleus (LGN: the thalamic nucleus in the visual pathway) is available in Horn et al. (2000); Sherman and Guillery (2001); and Jones (2007). Based on these thalamic and cortical physiological datasets as well as DTI (Diffusion Tensor Imaging) data obtained from two human samples, Izhikevich and Edelmann (2008a) have presented a comprehensive TCT circuit using minimal parameter spiking neural models (Izhikevich, 2003) to mimic spiking population behavior. The SpiNNaker-based TCT model presented here is at the level of abstraction of the model in Izhikevich (2003), and has two modules viz. a thalamic module and a cortical module. The design and layout of the thalamic module is as in Bhattacharya et al. (2011) and is based on physiological data obtained from Sherman (2006). The cortical module layout and parameterizations are based on a previous implementation on SpiNNaker (Sharp et al., 2012) that was designed to test fast, stable and power-efficient performance on SpiNNaker when compared with other available platforms. The detailed modeling approach and parameterizations is covered in section 2. To the best of our knowledge, we are not aware of any prior instance of mimicking EEG signals using the SpiNNaker machine; similarly, this is the first instance of implementation of a TCT model within the SpiNNaker framework.

In section 3, we present the preliminary results from this study based on our observation of the membrane potential time-series and power spectra of the cell populations. Specifically, the output of the excitatory cells of the thalamus and the cortical layer 4 are studied as a part of the first set of results from the TCT model simulation on SpiNNaker. An average of three trial runs of the model with all parameters at their initial values showed the membrane potential of both cell populations as noisy time series outputs with the dominant frequency of oscillation within the alpha band (8–12 Hz), a characteristic of quiet wakefulness. Next, we performed preliminary engineering of the model parameters to induce a sleep-wake transitional behavior in the model. The particular case we examined, which is outlined in more detail in section 3, was that of disconnecting the thalamic reticular nucleus (TRN) cell population in the model. This was designed to alter the thalamo-cortico-thalamic loop, which is responsible for the maintenance of the quiet wakefulness alpha rhythm, and simulate the situation during sleep in which cortical areas become functionally disconnected (Massimini et al., 2005). It thus provides a good test of the neuronal dynamics of the model in a situation in which the real dynamics are reasonably well understood. In previous (Bhattacharya, 2013; Bhattacharya et al., 2013) as well as ongoing (unpublished) work, lumped parameter models of neuronal population of the thalamocortical circuits [also known as neural mass models (Marreiros et al., 2009)] have shown dependence on the TRN connectivity for mimicking qualitative dynamics as seen in EEG patterns of sleep and quiet wakefulness. Our results showed some important similarities with real sleep EEG time series data (also shown) when the TRN population is disconnected. However, significant differences with sleep power spectral data have also been observed; this suggests the model requires further tuning before it can fully capture sleep/wake thalamocortical dynamics.

It is important to note that the purpose of the work presented here is to design a working model structure of the TCT circuit on SpiNNaker such that the model dynamics show some similarity to known dynamics of sleep and wake EEG in terms of characteristic spectral power; the intention is not to present a fully tuned model or a detailed exploration of those dynamics. A discussion on the motivation of the current work, the drawbacks, the implications of the initial results presented and future work plans is provided in section 4.

## 2. Materials and methods

In this section, we first give a brief background of the SpiNNaker architecture, followed by a detailed description of the TCT model and modeling methods adopted in this work. The simulation methods, and methods for observing results on the SpiNNaker platform are also outlined.

### 2.1. The SpiNNaker machine and tool chain

#### 2.1.1. The architecture

The SpiNNaker project, led by the University of Manchester and its partners in academia and industry, aims to create a biologically inspired high performance computing architecture for the simulation of large real-time Spiking Neural Networks (Furber et al., 2006, 2013). It incorporates characteristics of fault-tolerance and power frugality, similar to those of the biological brain, whose low-power and resilient performance is achieved through extensive parallel computation.

**Figure 1:**
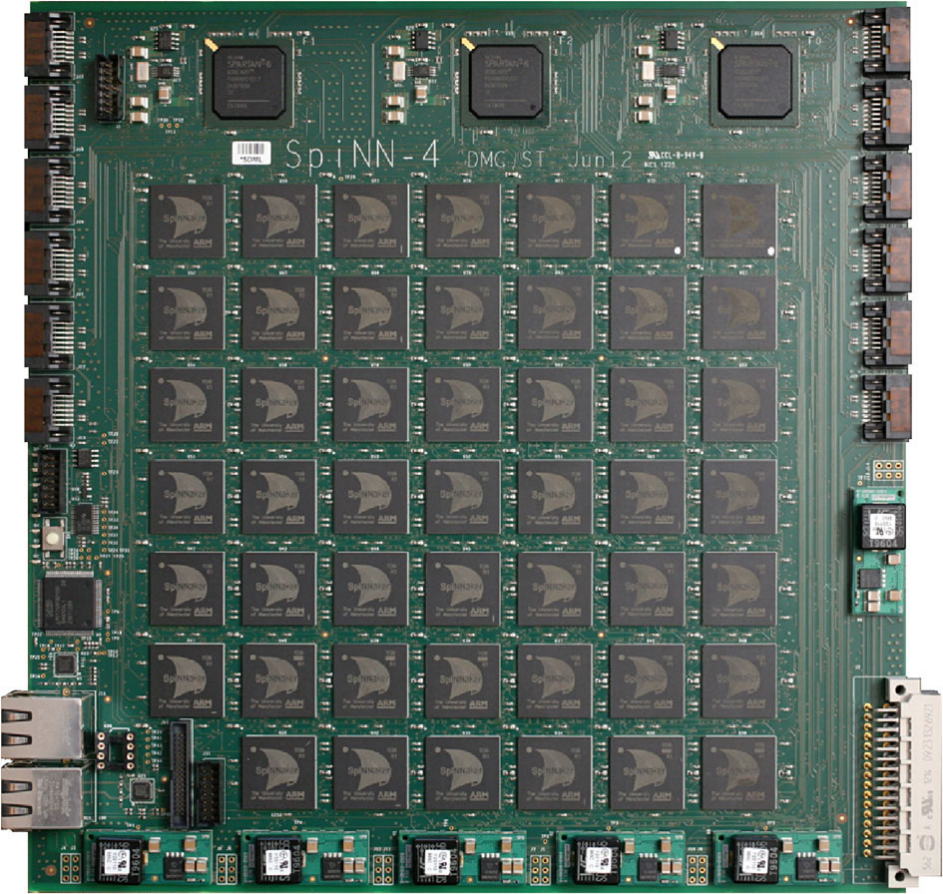
**A 48 chip SpiNNaker board (228 × 233 mm), the building block from which larger systems will be constructed**.

A SpiNNaker system is formed by the interconnection of SpiNNaker chips and boards (Figure 1), each chip being a custom Application Specific Integrated Circuit (ASIC) containing 18 ARM processors—the likes of which are found in mobile telephones. Each processor is low-power in operation, but fully programmable, permitting each to execute arbitrary neural and synaptic models. Spikes emitted by a simulated neuron in operation are conveyed as short packets to efferent neurons using a bespoke network on chip, and further afield to processors on neighboring chips using a network of connections which resiliently interconnect the chips to form the SpiNNaker machine.

The maximum number of chips in a SpiNNaker configuration is in excess of 65,000, and with 18 processors on each chip a machine can exceed one million processors. Even with the medium performance ARM processors used it is possible to simulate multiple neurons on each processor in real time, depending on their model complexity, potentially delivering many hundreds of millions of point-type neurons in a full deployment (Furber et al., 2006).

#### 2.1.2. Programming SpiNNaker

The selection of neuron and synaptic models and their interconnectivity is achieved by the user through a high-level modeling language. This flexible approach becomes increasingly important as networks grow in size, and it becomes impractical to specify each individual neuron and its connections—the network description is therefore made through multiple levels of hierarchy. The primary language used in the specification of Spiking Neural Networks to operate on SpiNNaker is PyNN (Davison et al., 2009), which is a popular description specification. Support of the PyNN library is enabled by a software tool-chain coined “PACMAN,” which has been developed to take this high level description of the network and perform Partitioning And Configuration MANagement (Galluppi et al., 2012). For example a 10,000 neuron network is analyzed by PACMAN, and partitioned into chunks which are manageable for a single processor using the neuron model specified. If each processor is able to handle 100 neurons of that type, then the partition size necessitates 100 processors and the tools take care of this partitioning and the necessary inter-connectivity. The next stage involves allocating the physical processors to this task based on the topology of the target SpiNNaker machine, the loading of data to it, and the execution and control of the simulation.

#### 2.1.3. Results recovery

There are two main methods of accessing the results on SpiNNaker. Firstly PyNN may be used to direct the simulation to make recordings of parameters periodically, for example neuron membrane potentials over time; and after the simulation this information may be recovered, processed and plotted. Secondly, it is possible to recover data from the simulation whilst it is “in-flight”—also requested through a PyNN parameter, for example to direct spike outputs to a “dummy” efferent neuron whose role is to collect and distribute spikes to an external receiver. This second method becomes particularly useful in simulations which run over an extended period, for example on a robot where a control loop is to be closed (Denk et al., 2013), or to simulate multiple channels of activity simultaneously, and to this end real-time visualization software (VisRT) has been developed (Patterson et al., 2012). In this study we make use of both methods, data is recovered post-simulation into MATLAB for analysis, and VisRT is used to gain an insight into the firing rates and rhythms seen in the simulations for EEG-type channel plots.

### 2.2. The thalamo-cortico-thalamic model

The TCT model has two modules: cortical and thalamic; all information on the model parameters are provided in Tables 2, 3. The thalamic module consists of the thalamocortical relay (TCR) cells, the inhibitory interneurons (IN) and the thalamic reticular nucleus (TRN). The synaptic connectivity layout and values of the thalamic module cell populations are sourced from Horn et al. (2000); Sherman (2006); and Jones (2007) and are as in our previous work (Bhattacharya et al., 2011). The cortical module cell populations are as described previously in Sharp et al. (2012) and are further subdivided into layers 2–6. Layer 1 is ignored in keeping with standard practice due to sparsity of neurons in this layer. Similarly, layers 2 and 3 are treated as a single layer in keeping with models based on physiology of the mammalian visual cortex (Binzegger et al., 2009). Each cortical layer consists of pyramidal (PY), basket (B) and non-basket (NB) cell populations. Layer 4 has an additional cell population of spiny-stellate (SS) cells.

The number of neurons in each cell population of the thalamic and cortical modules are provided in Tables 3B,C, respectively. The data on the proportion of cells of each type in the cortical layers are scaled versions of Izhikevich and Edelmann (2008a) and Sharp et al. (2012), which in turn are inspired by data from visual cortex of the cat as provided in Binzegger et al. (2004) and Douglas and Martin (2004). Based on literature reporting physiological data, it is estimated in Hill and Tononi (2005) that a thalamocortical column containing 94 (i.e., ≈100) neurons cover a surface area of 1454 μm^2^. The total number of cells in the TCT model is 1090 (i.e., ≈1000) and may therefore be thought to represent a column of interconnected neurons covering ≈0.15 mm^2^ of thalamocortical tissue.

Each synaptic connectivity parameter between two cell populations has two attributes: (1) a probability of connection *P* indicating the absence of all-to-all intra- and inter-module connectivity; and (2) the weight of the synaptic connectivity *C*, expressed as a percentage of the total number of synapses made on an individual synaptic node on the post-synaptic cell. In the cortical module, all *P* are identical to previous work (see Table 2, in Sharp et al., 2012) to ensure stability and comparability during simulation on SpiNNaker; the reader may refer to this work for details on how the specific values were obtained. All values for *C* in the cortical module are as in Izhikevich and Edelmann (2008a) and Sharp et al. (2012). In the thalamic module, and for connections between thalamic and cortical cells, the connection probabilities *P* are arbitrarily set to 0.25 for the sake of simplicity in this study. The intra-thalamic and corticothalamic values for *C* are sourced from previous work (Bhattacharya et al., 2011), which in turn are based on Horn et al. (2000) and Jones (2007). The values of *C* for the thalamocortical efferents to the SS and B cells of Layer 4 are sourced from Binzegger et al. (2004).

The TCR and IN cells of the thalamic module in the TCT model are fed with a spike source that follows a Poisson distribution with a spiking rate of 25 Hz and an all-to-all connectivity. The inter-module connectivities i.e., connections between the cortical module and the thalamic modules as well as between the external input source and thalamic module have an induced delay simulated by a uniformly distributed random number generator in PyNN.

### 2.3. Spiking dynamics of the thalamo-cortico-thalamic model neurons

Each neuron in the TCT model is an implementation of the spiking neuron model proposed in Izhikevich (2003), which is now a widely used template for modeling spiking neuron behavior due to its computational efficiency and rich dynamics, and is commonly referred to as the “Izhikevich model.” Our longer-term objective is to use the Izhikevich model to implement an appropriate spiking behavior for the neurons in each population of the TCT model based on experimental observations in biology. An excellent demonstration of how a changing set of parameter values in the Izhikevich model can simulate the various spiking dynamics of thalamocortical neurons is provided in Izhikevich (2004). We have adopted three types of spiking behavior in the model:

#### 2.3.1. Tonic spiking

Tonic spiking refers to a continuous train of spikes in response to an external stimulus and is known to be adopted by a cell when it is communicating information (McCormick and Feeser, 1990); for example tonic spiking of the TCR cells of the LGN indicate that they are in a “driver” mode and are passing retinal information to the visual cortex (Sherman, 2005). The tonic mode of spiking can be further classified based on a (qualitative) characteristic frequency of firing in response to a stimulus: regular spiking (*RS*) and fast spiking (*FS*). A comparison of *RS* and *FS* dynamics simulated using Izhikevich's model and from *in vitro* recordings on thalamocortical neurons is demonstrated in Izhikevich and Edelmann (2008a) (Figure 10 in the Supplementary Material of the cited work). We follow this work and parameterize the PY, SS and TCR populations in the TCT model to adopt similar *RS* dynamics in response to stimuli, while the cortical B cells are parameterized to respond in an FS mode. It may be noted that all the cell populations displaying the *RS* mode are excitatory in nature, while the inhibitory B cell population respond in a *FS* mode. For simplicity, we adopt a similar spiking behavior for the inhibitory IN cell population of the thalamus.

#### 2.3.2. Spike frequency adaptation

This terminology is used to define spiking dynamics where the inter-spike interval is low at the onset of the stimulus but “adapts” with passing time and the spiking frequency decreases. The cortical NB cells are modeled in Izhikevich and Edelmann (2008b) to exhibit a low threshold spiking (*LTS*) behavior, which is a type of spike frequency adaptation dynamics. We follow this work and parameterize the TRN cells in the TCT model to respond in an LTS mode to a step stimulus.

#### 2.3.3. Tonic bursting

Bursting behavior in neural dynamics refers to a series of spikes in quick succession; tonic bursting would thus refer to a train of such bursts of spikes. The burst spiking mode of the inhibitory TRN cell population is believed to be centrally important in generating the synchronized oscillations observed in EEG during slow wave sleep (Golomb et al., 1994; Destexhe and Sejnowski, 2002). The TRN cell population in the TCT model is parameterized to respond in a tonic bursting mode.

**Figure 2:**
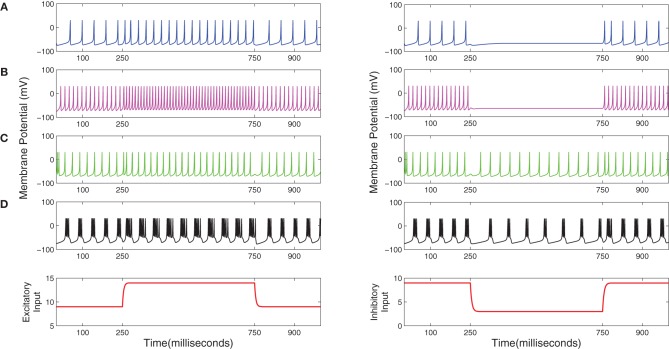
**(A)** Regular spiking *(RS)*, **(B)** Fast spiking *(FS)*, **(C)** Low threshold spiking *(LTS)*, and **(D)** Tonic bursting dynamics of the Izhikevich model (Izhikevich, 2003, 2004) simulated on the SpiNNaker chip using the PyNN interfacing software. Plots in the left (right)-hand-side column correspond to an excitatory (inhibitory) current stimulus applied between 250 and 750 ms during a 1000 ms simulation time.

All data used to parameterize the cell populations in the above-mentioned spiking modes is provided in Table 1 and based on the implementation of the Izhikevich model in Python by Galbraith (2011). The excitatory and inhibitory synaptic parameters are set by empirical study in PyNN corresponding to a set of parameters to simulate the desired spiking dynamics. The corresponding dynamics of a single example neuron in a population in response to an excitatory or inhibitory stimulus is shown in Figure 2.

**Table 1:** **The parameter set corresponding to the spiking dynamics shown in Figure 2**.

	**(dimensionless parameters)**				**(mV)**		**(ms^−^**1**)**		**(mA)**	**TCT model cells**
	**a**	**b**	**c**	**d**	**u**	**v**	τ^***E***^_***syn***_	τ^***I***^_***syn***_	**I**	
Regular spiking (*RS*)	0.02	0.2	−65	6	−60	0	5	6	9	PY, SS, TCR
Fast spiking (*FS*)	0.1	0.2	−65	6	−70	0	5	6	9	B, IN
Low threshold spiking (*LTS*)	0.01	0.2	−65	6	−70	0	5	6	25	NB
Tonic bursting	0.02	0.25	−50	2	−70	0	5	6	10	TRN

*All parameters are based on those provided in Galbraith (2011) for simulation using Python software. The final parameter values are adjusted by empirical study to simulate similar qualitative spiking dynamics on SpiNNaker*.

**Table 2:**
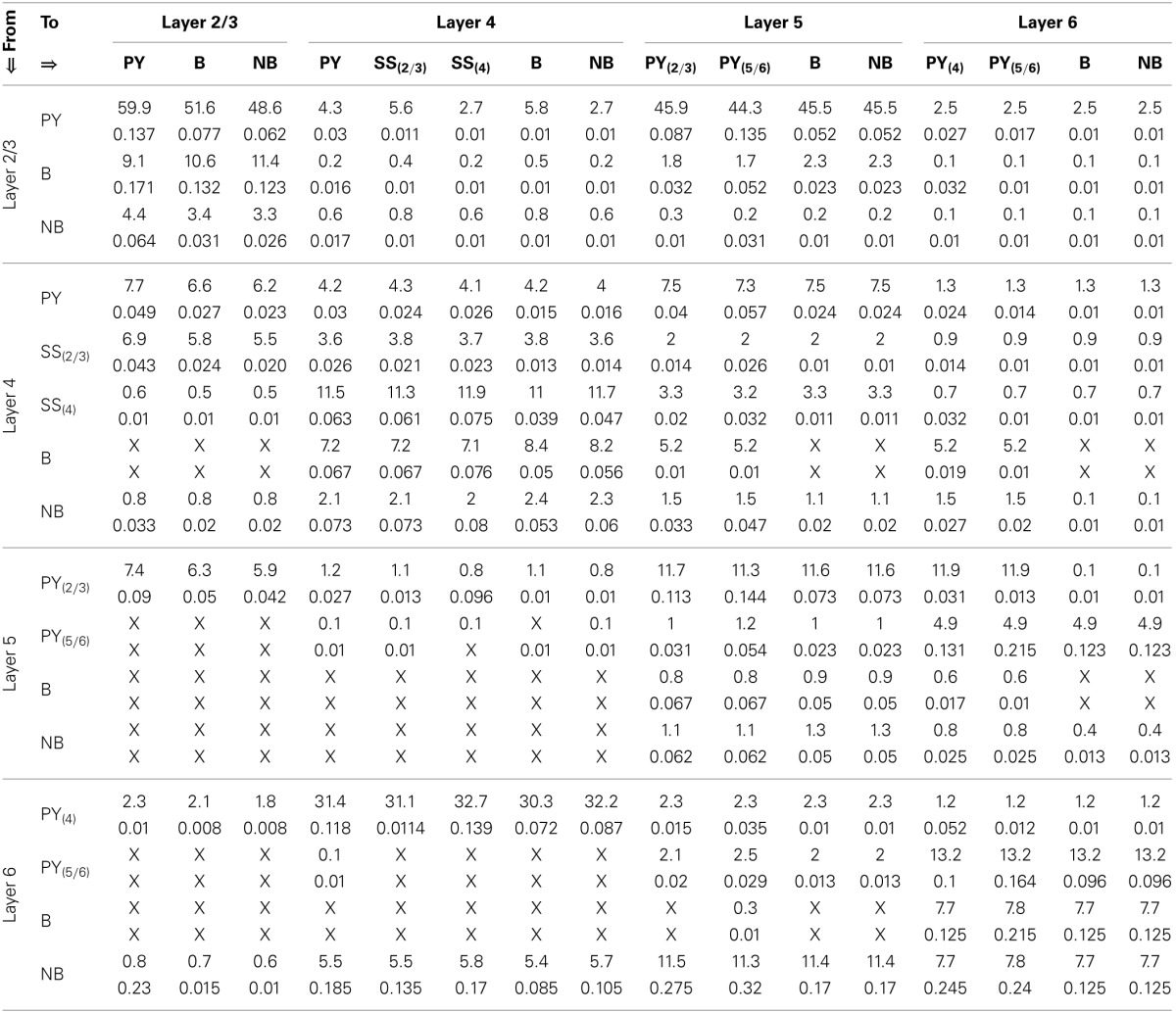
**The synaptic connectivity parameters between the cells of the cortical layers of the TCT model**.

## 3. Results

**Figure 3:**
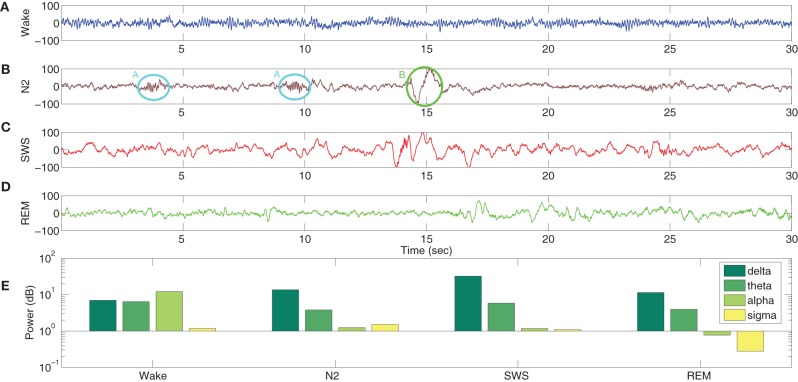
**EEG characteristics of human sleep and wake**. Quiet wakefulness is represented in panel **(A)** and is characterized by the presence of the alpha rhythm, which is absent during sleep (see the power spectra at the bottom of the figure). Normal sleep, often referred to as N2 in sleep literature, is represented in panel **(B)** and is characterized by the presence of spindles (A, circled in cyan) and K-complexes (B, circled in green). Slow wave sleep (SWS) is represented in panel **(C)** and is characterized by high amplitude slow oscillations. REM sleep **(D)** has a mixed frequency pattern, and is additionally identified by the presence of eye movements and low muscle tone. The power spectra in the four bands involved in distinguishing wake and different stages of sleep **(E)** shows a greater delta power during the sleep stages, while quiet wakefulness has stronger alpha power. Data taken from Durrant et al. (2013).

A typical human EEG recording taken during quiet wakefulness and sleep (Durrant et al., 2013) is shown in Figures 3A–D. Sleep in birds and mammals is divided into REM (Rapid-Eye-Movement) and non-REM parts. Non-REM sleep is further divided into light/transitional sleep (N1), which makes up 5–10% of the night and is not considered functionally significant; normal sleep (N2; Figure 3B), which is characterized by the presence of spindles and K-complexes and is present for 40–50% of the night; slow wave sleep (N3/SWS; Figure 3C) which is the deepest form of sleep and characterized by the presence of high-amplitude low-frequency (“slow”) waves. REM sleep (Figure 3D) is characterized by a mixed frequency waveform, low muscle tone and rapid eye movements. Sleep EEG is classified into these different stages based on 30 s epochs according to standardized sleep scoring criteria (Rechtschaffen and Kales, 1968; Ancoli-Israel et al., 2007). As a complement to the characteristic waveforms, power spectral density also differs considerably between sleep stages (Figure 3E). In particular, spectral power in sleep and quiet wakefulness is generally analyzed in four bands: delta (1–4 Hz), theta (4–8 Hz), alpha (8–12 Hz), and sigma (sometimes called the spindle band; 12–16 Hz). Higher frequencies in the beta and gamma ranges are associated with active wakefulness and task completion and are not involved in identifying sleep or wake patterns; these bands are not considered further here. In Figure 3E, the power spectra in all the sleep stages (REM and non-REM) are dominated by the delta band. In contrast, the power spectra in quiet wakefulness is dominated by the alpha band.

In order to test the ability of the model to capture some basic neuronal dynamics, we ran simulations and compared the model output to the recorded EEG data in Figure 3. The average membrane potential of all neurons in each cell population of the TCT model is considered as the output membrane potential of the population. Although EEG is believed to represent dendritic post-synaptic potentials from pyramidal neurons in the cerebral cortex, the TCR cell output in thalamocortical population models have been shown to mimic alpha rhythmic and slow-wave EEG characteristics (da Silva et al., 1974; Suffczyński, 2000; Bhattacharya et al., 2013). Along these lines, in this work, we focus on the TCR cells of the thalamic module and the main target of their efferents to the cortical module (Gil et al., 1999; Lee and Sherman, 2008) viz. the Pyramidal cells in Layer 4 (PY4). Recent studies (Crunelli et al., 2011; Crunelli and Hughes, 2012) have identified the central role of the inhibitory neurons of the TRN acting via the TCR neurons in generating both slow oscillations and spindles that characterize non-REM sleep. In previous work, we have shown the pivotal role of the TRN cell afferents in effecting a time-series bifurcation of the TCR cell output in a population model of the thalamocortical circuit (Bhattacharya et al., 2013). In this work, we present a preliminary test on the TCT model by studying the output time series and power spectra with all model parameters at their base values. We then compare this with the case when the TRN cell population is disconnected from the model.

**Figure 4:**
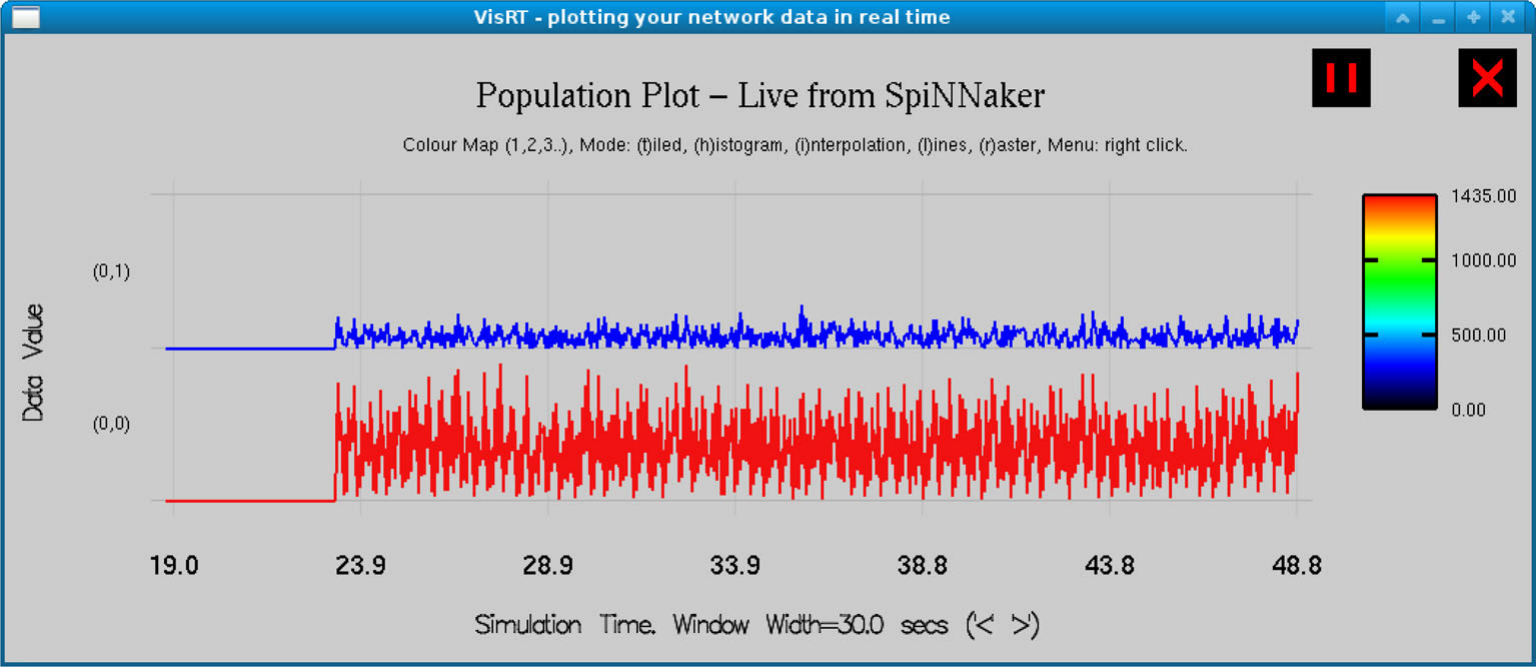
**Real time output from the simulation of the TCT model on the SpiNNaker board as observed in visRT**. The spiking rate of the TCR (top) and PY4 (bottom) populations for each period of 25 ms over a total simulation time of 30 s is shown.

The model is simulated on SpiNNaker for 30 s for each simulation at a resolution of 1 ms, and subsequently downsampled to 200 Hz. The mean membrane potential of the PY4 and TCR cell population are averaged across three simulation runs to improve the reliability of the results. A snapshot of the real-time visualization of the model simulation on SpiNNaker as seen using visRT is shown in Figure 4. The human EEG used for comparison is recorded at 200 Hz from an occipital electrode (O1) referenced against the contralateral mastoid. Sleep stages are independently classified by two experts with more than 90% agreement. Both human EEG and the model output are filtered between 1 and 16 Hz with a Butterworth bandpass filter of order 10 in order to focus on spectral bands of interest. Power spectral density is estimated using a Welch periodogram with 800 FFT points using a Hamming window half the length of the sampling frequency and a 50% overlap.

**Figure 5:**
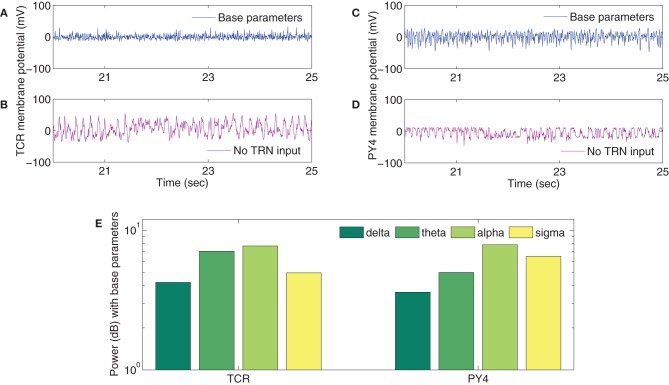
**Sample of the time series outputs of the **(A,B)** TCR and the **(C,D)** PY4 cell populations for a period of 5 s, clipped arbitrarily between the 20th and the 25th s from the 30 s (unfiltered) signal and downsampled to 200 Hz**. A comparison with real EEG time series data of quiet wakefulness (Figure 3A) shows a similarity with the **(A)** TCR and **(C)** PY4 outputs when all model parameters are at their basal values. A comparison with real EEG time series data of SWS (Figure 3C) shows a similarity with the **(B)** TCR and **(D)** PY4 outputs when the TRN cell population is disconnected from the model. **(E)** The power spectra of the TCR and PY4 cell populations with all model parameters at their basal values. A dominant alpha rhythm is observed, similar to that in the real EEG power spectra of quiet wakefulness (Figure 3E). (The reader may kindly note that the results presented here is a preliminary attempt in studying the plausibility of simulating EEG rhythms in models developed on the SpiNNaker computer. At no point do we expect to see exact match of model results with real EEG data; rather, we do expect to identify differences between the two that will inform our ongoing work).

The TCR time-series output with all model parameters maintained at basal values (Figure 5A) show a similarity with the EEG time series in quiet wakefulness (Figure 3A). The corresponding time series output of the PY4 cells are shown in Figure 5C and show a similarity with their main “driver” cells of the TCR, albeit with a larger amplitude of oscillation. It may be noted that the time series plots presented in Figure 5 are unfiltered data sampled at 5 ms intervals (200 Hz). A power spectra analysis of both the TCR and PY4 outputs corresponding to basal parameters show a dominant frequency within the alpha band (Figure 5E), similar to the power spectra of quiet wakefulness shown in Figure 3E. Next we disconnect the TRN cell population from the TCT model by removing the connectivity from the TRN to the TCR and vice-versa (see Table 3A). We note a distinct bifurcation in both the TCR and PY4 time series output shown in Figures 5B,D, respectively with a reduced frequency of oscillation compared to the output corresponding to basal parameters; an increased amplitude of oscillation is also observed in the TCR output (Figure 5B). A comparison of the TCR time series with real EEG data show a resemblance with the SWS time series (Figure 3C). However, the frequency of the oscillatory activity in Figures 5B,D appears (on visual inspection) to be higher than that in Figure 3C. This observation is reflected in the power spectra of both TCR and PY4 cell populations corresponding to disconnection of the TRN, showing a dominant frequency within the theta band (not shown here). This is unlike the power spectra of SWS, which have a dominant frequency within the delta band. Further, we observe that the amplitude of oscillation in the PY4 output time series does not show any significant increase with TRN disconnection, which is not in agreement with the classic definition of EEG “slowing” (reduced frequency, higher amplitude).

**Table 3:**
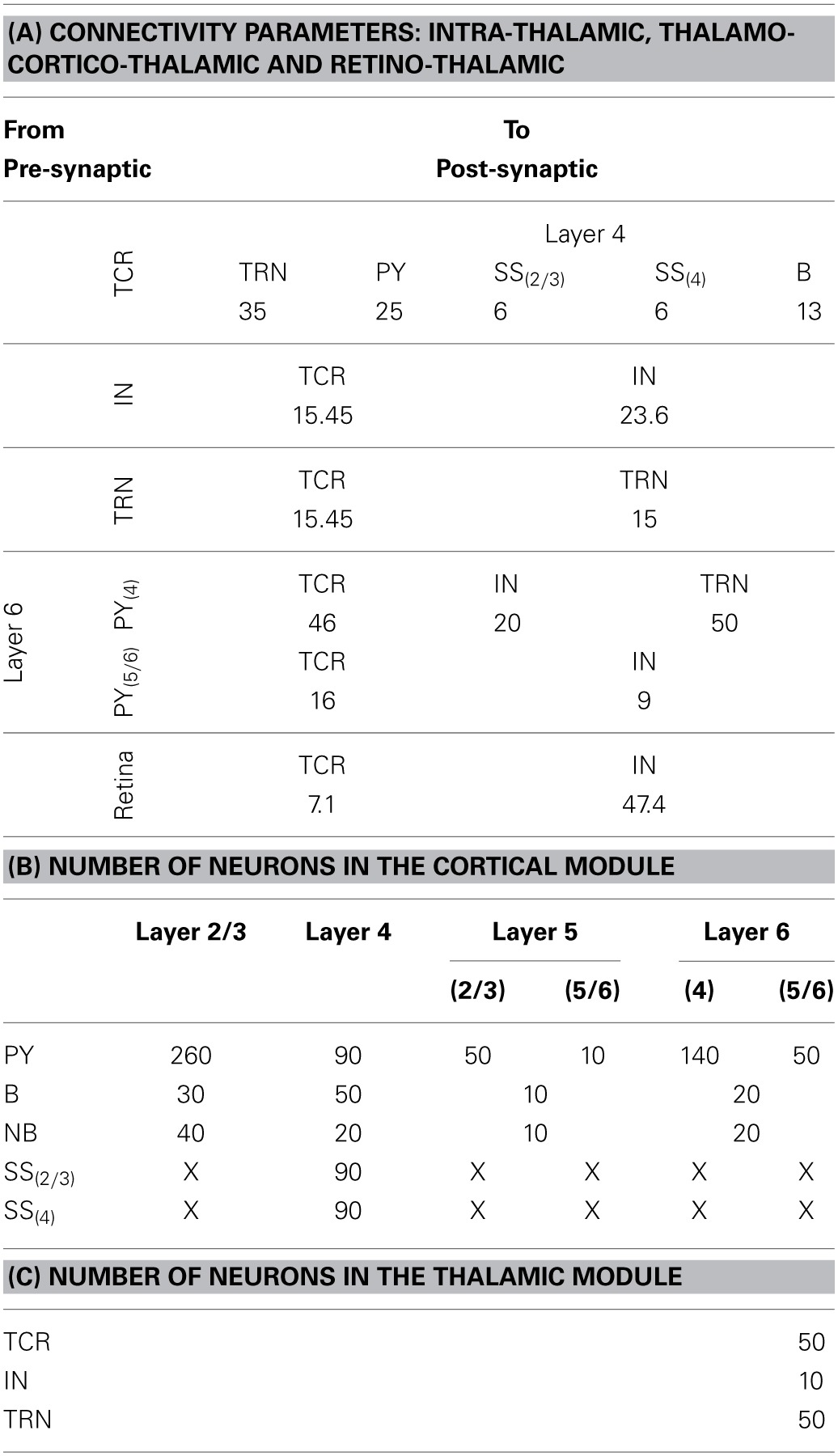
**(A) The “weight” of the synaptic connectivities between the thalamic and cortical module cells as well as between thalamic cell populations. The probability of connection for inter-module connectivity is 0.25 in the current model. The synaptic connections from the retina to the thalamic cells have an all-to-all connectivity. (B) The population of neurons of each type in the cortical module are mentioned in the first column and the cortical layers are mentioned in the top row. The cortical layer references within brackets (for Layers 5 and 6 and for the SS cells) indicate the dendritic arborization of the cells to these layers. An “X” indicates the lack of the cell type in the cortical layer. (C) The population of neurons of each type in the thalamic module**.

Overall, and given the preliminary nature of this work, we would not expect the model parameters to be tuned to give a perfect replication of human EEG, and indeed we do see substantial differences between the two. The most important difference between the model output and human EEG at present is the lack of strong delta power with the TRN cells disconnected from the model, and this area should be prioritized for further research.

## 4. Discussion

Sleep and its biological relevance and mechanisms have been of interest in research (Rasch and Born, 2013) and beyond; a “healthy” sleep pattern have tremendous impact on daily activities (Mednick and Ehrman, 2006). Thus it is not surprising that sleep disturbances are a common accompaniment of several neurological and psychiatric disorders (Brown et al., 2012). Additionally, the time and frequency signatures of sleep electroencephalography (EEG) in neurological disorders often provide a better understanding of the disease conditions [for example in schizophrenia (Gardner et al., 2014); Alzheimer disease (Jonkman, 1997)]. Furthermore, rapid-eye-movement (REM) sleep is thought to play a role in memory consolidation involving the non-hippocampal brain parts (Born et al., 2006). The thalamo-cortico-thalamic circuitry plays a key role in generating brain rhythms (Steriade et al., 1993; McCormick and Bal, 1997). Several studies on thalamocortical dynamics have used mesoscopic scale lumped parameter models to mimic EEG in healthy conditions (Robinson et al., 2002; Zavaglia et al., 2006; Deco et al., 2008; Modolo et al., 2013; Moran et al., 2013), as well as to investigate anomalous EEG in neurological disorders (Suffczyński et al., 2004; Roberts and Robinson, 2008; Pons et al., 2010; de Haan et al., 2012). In recent research (Bhattacharya, 2013), which is along similar lines as in Lytton (1996); Erdi et al. (2006), the need for detailed synaptic mechanisms in thalamocortical lumped parameter models to facilitate biologically realistic mapping of model features is emphasized. While extended work on the lumped parameter model implementing synaptic dynamics remains ongoing, we believe it is necessary to have a parallel line of investigation using a population model comprising of network(s) of single neuron models (i.e., single-neuron-level population model as opposed to lumped parameter population models) that is similar in structure to the former. This gives a “two-scale” architecture to the thalamo-cortico-thalamic framework. The endeavor will be to use the framework for realistic simulation of EEG dynamics in sleep-wake transition. Here, we have presented a preliminary study on inducing a transition from quiet wakefulness to a “slow wave” (higher amplitude, lower frequency) pattern in the model output, and have shown the similarity and dissimilarity of the model output with real EEG data of sleep and wakefulness; these are discussed further below.

The primary issue in building a single-neuronal-level population model is the deficiency in available computational resources in terms of implementing biologically plausible parallel and asynchronous information transmission and exchange within the model framework. Another key aspect is energy-efficiency whereby maximal information processing is carried out using minimal resources, a mechanism that allows biology to deal with massive amounts of data in a fast and power efficient manner. This necessitates specialized computational tools to provide a low-power, parallel asynchronous framework for building very-large-scale-biologically-plausible models (VLSBm). The SpiNNaker (Spiking Neural Network architecture) chip is a platform designed to occupy this space; it meets all of the above criteria for building VLSBm and has been tested to outperform current available software and hardware platforms when building a cortical model of spiking neural networks (Sharp et al., 2012).

In this work we have built a thalamo-cortico-thalamic spiking neural network for implementation on SpiNNaker. The mini-framework consists of 1090 neurons to mimic approximately 0.15 mm^2^ of thalamocortical tissue. We have focussed on the thalamocortical relay (TCR) cells and the cortical Layer 4 pyramidal (PY4) cells; the layer 4 cells are known to be dominated by the sensory pathway input from the thalamus compared to inputs from other cortical areas (Gil et al., 1999). With all model parameters at their base values, the TCR time series output and its power spectra resembles the EEG characteristics of quiet wakefulness. Observation of the corresponding PY4 cell outputs indicate that the behavior of these cells are largely driven by the TCR cells. Next, we endeavored to vary specific model parameters to simulate non-rapid eye movement (non-REM) sleep stages. The thalamic reticular nucleus (TRN) neurons are implicated in playing a vital role in effecting slow wave oscillation in the EEG such as observed during slow wave sleep (SWS). To test this feature in the model, we disconnect all efferents from and afferents to the TRN cell population. We observe a distinct transition in the time series behavior of both the TCR and PY4 cells that resemble the EEG time series in SWS, albeit at a slightly higher frequency of oscillation (observed by visual inspection). This observation is reflected in the power spectra where the dominant frequency of oscillation for both population outputs are within the theta band, unlike the dominant delta band frequency seen in all stages of sleep EEG data. We speculate that the current disagreement in the power spectra of the SWS simulation on the TCT model may be addressed by dynamically changing the spiking behavior of the model cell populations (see below for further discussion on this). Furthermore, it will be interesting to observe how the intracortical afferents affect the PY4 cells in comparison to the TCR afferents (Destexhe, 2008; Lee and Sherman, 2008) and whether the model behavior conforms to experimental observations. Nonetheless, we note that the framework presented herein is a pilot study only, designed primarily to test the ability of the hardware to capture thalamocortical dynamics. We believe that the outcome from this study will provide a “basis” for simulating EEG signals on SpiNNaker-based computational models. Thus, at this stage, we do not attempt to simulate a true replication of the sleep-wake dynamics on the model. The larger goal of the work is to lay the foundations for building a VLSBm of thalamocortical interactivity to simulate biologically realistic sleep rhythms as observed in EEG. However, further testing and simulation on SpiNNaker will be required before scaling up the model for realistic simulation of EEG rhythms; we will take this up as an extension of the current work. Altogether, we believe this is a promising first demonstration of SpiNNaker as a platform for investigating thalamocortical circuits in humans.

A widespread current concern in the computational neuroscience community is the non-trivial task of populating the parameter space of computational models; the task gets harder with increasing model size as experimental data with definitive values for specific parameters are difficult to acquire. We have sourced appropriate model parameter values from Binzegger et al. (2004); Izhikevich and Edelmann (2008b); Bhattacharya et al. (2011); Galbraith (2011); and Sharp et al. (2012). Model layout and neuronal dynamics are from Sherman (2006) and Bhattacharya et al. (2011) and Izhikevich (2003, 2004), respectively. The absolute values of the model parameters often require appropriate scaling for the simulation platform, and a common approach to deal with this aspect has been to normalize all model parameters to a “simulator-friendly” scale. Along these lines, several assumptions and simplifications have been made in this study:

First, burst spiking dynamics of the thalamic cells that are crucial for generating slow wave oscillations (Jeanmonod et al., 1996; Magnin et al., 2005) are explored minimally. The thalamo-cortical relay (TCR) cells are tested for tonic spiking behavior in this work, which best align with the awake state of the brain. We speculate that the results reflect this behavioral mode of the TCR cells, clearly showing a resemblance with both time-series and power spectra of EEG in quiet awake state. However, the TCR displays burst spiking dynamics during the stages of sleep. Similarly, the TRN cells are known to show rich spiking dynamics (e.g., rebound bursting, low threshold spiking) that underlie sleep-wake oscillatory activity. These variant dynamics of the TCR and TRN cells will be further investigated in our ongoing work. Thalamic interneurons are more problematic; there are to our knowledge no references in the modeling literature relating specifically to the spiking dynamics of the thalamic interneurons (Destexhe et al., 1998). However the cortical basket cells, which are also categorized as local interneurons depending on their function and dendritic arborization, are described in Izhikevich and Edelmann (2008a) using Fast Spiking (FS) dynamics. We have arbitrarily adopted this spiking behavior for the IN cells. Overall, much more detailed exploration and simulation of the individual thalamic cell spiking dynamics needs to be performed to preview the parameter space that would allow full replication of EEG in different sleep stages and the sleep-wake transition. It needs to be mentioned here that a high number of synaptic efferents from the thalamic interneurons are dendro-dendritic (Cox and Sherman, 2000). However, this aspect does not affect the synaptic transmission in the TCT framework as it comprises of spiking neuron models, and does not take into account the detailed axonal and dendritic attributes related to spike transmission and reception.

Second, the Izhikevich model uses common excitatory and inhibitory synaptic parameters for all cell populations of excitatory and inhibitory types. This is a significant limitation and requires modification in future versions of the model to enable a direct comparison with the current lumped parameter models that include neurotransmitter and receptor dynamics.

Third, the neuronal population in the thalamus represents a loose estimate as no definitive data on the number of thalamic cells within a cortical column is available from literature. We preserve the (intra-thalamic) proportion of thalamic cells in the (Izhikevich and Edelmann, 2008a) thalamocortical model (only “specific nucleus” parameters are considered; the “non-specific nucleus” parameters are ignored), but scale this up by a factor of 10^2^. This may be contrasted with a factor of 10 scaling of the number of cortical cells. Thus the model is designed to place increased emphasis on the thalamic behavior and its effects on cortical oscillations for our test purposes.

Fourth, our objective is to simulate EEG in sleep and quiet wakefulness. Thus, the simulated retinal input to the model needs to conform to discharge rates of the retinal spiking neurons during the resting state. In an early work on the cat retina (Kuffler, 1953), it is observed that the resting state discharge rate of a single retinal neuron is approximately 25 Hz. This is in agreement with the spike source rate provided as input to the TCT model in this work. However, in a relatively recent work (Robinson et al., 2004), it is estimated that the resting state firing rate of retinal input is 11 Hz, while in an alert awake state this is in the range 12–20 Hz. Thus, it would need further work to test these variations in experimental data and the effects on the model output in context to mimicking sleep-wake EEG.

Fifth, the probability of connection between the intra-thalamic cells as well as for the feedforward and feedback connections between the thalamus and the cortex is arbitrarily set at 0.25 by empirical study on SpiNNaker. This will need further attention and more detailed tuning in future work.

Finally, the conduction delay for thalamocortical and corticothalamic communication is implemented using a uniformly distributed function to generate a random delay. However, data acquired from physiology and tested on computational models is available in literature (Roberts and Robinson, 2008). This will be explored for implementation in future work.

In conclusion, we have presented a pilot study which involved building biologically plausible networks on a biologically plausible computational platform—SpiNNaker. The study examines the feasibility of simulating EEG rhythms of sleep and wakefulness by implementing a thalamo-cortico-thalamic framework. The longer-term aim is to build a VLSBm of thalamo-cortico-thalamic synaptic interactivity on SpiNNaker, which will then be validated with real EEG data collected during sleep (Durrant et al., 2013). The work presented here gives a preliminary study of this approach. Ongoing work to build a similar framework with the lumped parameter approach will provide a “multi-scale” architecture to the model in both space and time. Together these models should provide new insights into the mechanisms which give rise to the rich thalamocortical dynamics seen in the human brain.

### Conflict of interest statement

The authors declare that the research was conducted in the absence of any commercial or financial relationships that could be construed as a potential conflict of interest.
